# Emergency Department Alternatives to Opioids: Adapting and Implementing Proven Therapies in Practice

**DOI:** 10.3390/ijerph20021206

**Published:** 2023-01-10

**Authors:** Sarah B. Floyd, Sam NcGarby, Susan Cordero Romero, Sam Garrison, Kevin Walker, William Hendry, Phillip C. Moschella

**Affiliations:** 1 Department of Public Health Sciences, Clemson University, Clemson, SC 29631, USA; 2 Department of Emergency Medicine, Prisma Health-Upstate, Greenville, SC 29605, USA; 3 Division of Pain Management, Prisma Health-Upstate, Greenville, SC 29605, USA; 4 Integrated Health Partners, Greenville, SC 29609, USA

**Keywords:** opioid, analgesic, emergency medicine, emergency department, pain management

## Abstract

The use of opioids to treat pain can increase the risk of long-term opioid dependency and is associated with negative patient outcomes. The objective of this study was to present the initial results following the implementation of Emergency-Department Alternatives to Opioids (ED-ALTO), a program that encourages the use of non-narcotic medications and procedures to treat pain in the Emergency Department (ED). We used a pre- and post-implementation study design to compare in-ED opioid utilization, as well as ED-ALTO medication and procedure use in the year before and after the program’s implementation. After ED-ALTO’s implementation, there was a decrease in opioid utilization in the ED and an increase in ED-ALTO medication use. Additionally, there was an increase in ED-ALTO procedure utilization and the complexity of conditions treated with ED-ALTO procedures, including the use of regional nerve blocks for shoulder dislocations and hip and rib fractures. In 8 of the 12 months following ED-ALTO’s implementation, a lower proportion of patients receiving ED-ALTO procedures received an opioid, and the opioid dosage was lower compared to patients with the same diagnoses who received standard care. The continued expansion of ED-ALTO programs across the US may serve as a mechanism to reduce opioid utilization and safely and successfully treat pain in ED settings.

## 1. Introduction

Pain is one of the most common reasons patients seek emergency medical care; accordingly, pain-related complaints account for up to 42% of all Emergency Department (ED) visits in the United States [1,2]. In order to function in a fast-paced clinical environment, ED physicians utilize opioids for one out of every three patients cared for in the ED, with the aim of quickly and effectively treating a patient’s pain [1]. However, the use of opioids to treat pain can increase the risk of long-term dependency [3] and carries negative risks to a patient’s overall health and long-term outcomes [4,5]. Currently, in the United States, there are no regulations on the usage of opioids in acute clinical settings. As ED physicians strive to quickly manage patients’ pain-related complaints, alternative solutions for treating pain safely and effectively in the ED are needed [6].

Innovative programs that encourage the use of non-narcotic medications and procedures to treat pain in the ED have recently been evaluated [7,8,9,10,11]. These programs, collectively known as Emergency-Department Alternatives to Opioids (ED-ALTO) programs, have shown initial promise with respect to reductions in opioid use [7,8], effective pain control, and patient satisfaction [7,8,9]. The early ED-ALTO programs primarily focused on the adoption of non-narcotic pharmacologic therapies to treat pain [7,8,9]. However, the additional adoption of proven procedures from other medical specialties to treat pain in the ED setting offers great potential regarding the treatment of high-frequency, low-acuity pain complaints. For example, nerve blocks can serve as important opioid-free adjunct therapies within ED-ALTO regimes. The use of femoral and fascia iliaca nerve blocks have been associated with significant reductions in the use of intramuscular and intravenous opioids in the ED [12,13], and the use of suprascapular nerve blocks for shoulder reduction have shown a decrease in pain and time spent in the ED [14]. The use of erector spinae blocks in the ED have also demonstrated efficacy in reducing pain complaints in patients suffering from rib fractures [15]. In addition, studies suggest that trigger point injections [16,17,18], acupuncture [19], and electrical nerve stimulation [20,21] can effectively treat musculoskeletal pain, highlighting the potential of these therapies in terms of providing pain control for patients visiting the ED.

While several published studies have demonstrated success through the use of ED-ALTO programs, no studies have evaluated ED-ALTO programs utilizing a combination of both medication- and procedure-based approaches to the treatment of pain. The objective of this study is to present the initial results of the implementation of a multi-faceted ED-ALTO program at a Level I Trauma Center ED located in the Southeastern US that utilizes alternative medication- and procedure-based approaches to manage pain in the ED. This program includes systematic training to create a culture of change within our ED and create sustainable, opioid-free treatment options to help treat various pain complaints commonly cared for in the ED.

## 2. Materials and Methods

### 2.1. Study Design and Setting

The ED-ALTO study was implemented on 1 August 2021, within a large, Level 1 Trauma Center in South Carolina in the Southeastern part of the United States. This research implemented a pre- and post-implementation study design comparing in-ED opioid utilization as well as ED-ALTO medication and procedure use. Data collection took place 12 months before the intervention and 12 months following implementation to reduce seasonal variability. The primary study location’s ED is a regional tertiary care referral center for the largest health system in the state. The ED sees approximately 100,000 adult patients a year and contains a three-year emergency medicine residency program with thirty residents. This study was approved by the Prisma Health-Upstate Institutional Review Board.

### 2.2. Study Eligibility and Enrollment

English-speaking adult patients presenting to the ED with pain-related conditions were eligible for ED-ALTO treatment. Our intervention targeted low-acuity pain conditions; therefore, patients with an Emergency Severity Index (ESI) below 3 were not eligible. The ESI is a triage tool that uses five levels from 1 (most urgent) to 5 (least urgent) to estimate the severity of illness and resource needs. Additionally, patients who were incarcerated or otherwise unable to provide informed consent to participate were excluded. Study personnel were on-site in the ED one day a week to identify and enroll eligible patients for ED-ALTO medication and procedure use. On enrollment days, study personnel monitored the ED track-board in real time for patients arriving with a pain complaint or were contacted by an ED physician for a patient consult within the electronic health record (EHR) system. If the patient was deemed eligible, the study coordinator would approach the patient to explain the study and ask for their consent to participate.

### 2.3. ED-ALTO Medications and Procedures

ED-ALTO therapies included the use of alternative medications and procedures to treat pain. A specific order set containing various medication choices based on a previous publication [8] was added to our hospital formularies under an “ALTO Study ED Pain Management” label within the EHR. This order set is categorized by types and areas of pain complaints, namely, headache/migraine, musculoskeletal, renal, abdominal, and extremity fracture/joint dislocation. Medication options are further delineated into first-line, second-line, and alternative options if applicable (see Appendix A, Table A1). ED-ALTO procedures included nerve blocks, trigger point injections, transcutaneous electrical nerve stimulation (TENS), and acupuncture. Acupuncture was offered alone or in conjunction with TENS or Percutaneous Electrical Nerve Stimulation (PENS). Three primary nerve blocks and one secondary nerve block were identified as applicable in the emergency setting to help treat pain from shoulder dislocations, hip fractures, and rib fractures. These included suprascapular, fascia iliaca, and erector spinae plane nerve blocks as primary blocks, as well as an axillary nerve block that pairs with the suprascapular block for shoulder anesthesia.

### 2.4. ED-ALTO Training and Education

Physicians’ education with respect to ED-ALTO medications and procedures was provided through multiple channels, including (1) direct bedside training, (2) live lectures/simulation education, and (3) asynchronous recoded video lectures, simulation sessions, and reading materials. Direct bedside training was provided on our scheduled “Opioid-free days”, which occurred one day a week. The goal of “Opioid-free days” was to encourage the use of ED-ALTO therapies on these days and reduce opioid prescription. On “Opioid-free days”, direct bedside support, training, and education was provided by two physicians: the Division Head of Anesthesia and Pain Management for the health system (K.W.) and a local practitioner of Acupuncture and Oriental Medicine (W.H.). On the “Opioid-free days”, these physicians were available from 10 a.m.–6 p.m. to provide direct procedural support and bedside training to the attending ED physicians, resident physicians, physician assistants, and nurse practitioners performing ED-ALTO procedures for patients.

### 2.5. Measurement and Statistical Analysis

Data were collected for 12 months pre-implementation from 1 August 2020, to 30 July 2021, and for the same 12-month period after implementation, which was from 1 August 2021, to 30 July 2022. Data were provided from the EHR, Epic^®^ (EPIC Systems, Madison, WI, USA), and included patient demographics, use of ED-ALTO medications, procedures, and opioid utilization. Encounter-level data elements included date of service, principal visit diagnosis, age, payer status, disposition, and patient sex. T-tests and Chi-square tests were performed to assess differences in the pre- and post-implementation samples as well as the utilization of opioids and ED-ALTO medications. Statistical significance was set at *p* < 0.05. The frequency of ED-ALTO procedure use was reported by diagnoses groups for the pre- and post-implementation periods.

We conducted a matched analysis in the post-intervention period. We found all diagnoses codes for patients receiving ED-ALTO procedures and matched them to patients with the same diagnoses that did not receive ED-ALTO procedures and received standard care (see Appendix A, Table A2). In the matched groups, we described and compared opioid prescription rates and morphine milligram equivalents (MME). All statistical analysis was performed using SAS version 9.4 (SAS Institute Inc., Cary, NC, USA).

## 3. Results

### 3.1. Characteristics of the ED Population

The patients presenting to the ED in the pre- and post-implementation periods were not meaningfully different. On average, the patients presenting to the ED were 51 years of age and 50% were female. The majority of patients were White (62%) or Black (27%), and 50% were publicly insured, followed by 26% of the sample that had private health insurance. In the post-implementation period, a slightly higher proportion of patients had public insurance (51.4% vs. 49.6%; *p* < 0.01) and a lower proportion were admitted to the hospital directly following their ED visit (20.5% vs. 22.0%; *p* < 0.01). In general, the ED patient population appeared similar in the pre- and post-implementation periods. Table 1 contains a detailed comparison of select patient characteristics across time periods.

### 3.2. ED-ALTO Medication and Opioid Utilization

Table 2 displays ED-ALTO medication and opioid utilization in the pre- and post-implementation periods. The utilization of ED-ALTO medications significantly increased from the pre- to post-implementation periods, with 33.6% of patients in the ED receiving ED-ALTO medication prior to implementation and 34.5% of patients in the ED receiving ED-ALTO medication after the program’s implementation (*p* < 0.01). The overall utilization of opioids decreased in the post-implementation period from 17.7% to 16.5% (*p* < 0.01), with significant reductions in the use of Dilaudid (2.7% to 1.6%; *p* < 0.01) and Hydrocodone (3.3% to 2.9%; *p* < 0.01). Among the patients receiving opioids, the average MME received increased in the post-implementation period (20.3 MME to 21.3 MME; *p* < 0.01).

### 3.3. ED-ALTO Procedure Utilization

In the pre-implementation period, a total of 98 ED-ALTO procedures were performed. After implementation, the total frequency of ED-ALTO procedures performed was 167. Prior to the ED-ALTO program’s implementation, neither acupuncture nor electrostimulation, either through TENS or PENS, had ever been utilized in this ED. After implementation, acupuncture was used to treat joint pain, lower back pain, and abdominal pain. Similarly, the use of trigger point injection was increased in the post-implementation period and its use was also extended outside of the treatment of headaches to include abdominal and back pain.

The use of nerve blocks saw an expansion in both overall frequency and clinical condition complexity, with an expanded use of the more complex regional nerve blocks. In the pre-intervention phase, digital nerve blocks were the primary nerve blocks used. These are commonly used for injuries of the hand, wrist, or fingers. Suprascapular, axillary, fascia iliaca, and erector spinae plane regional nerve blocks were all introduced as part of this program and used in the post-implementation period to treat fractures of the femur/hip and ribs as well as shoulder dislocations. Table 3 displays the ED-ALTO alternative procedure utilization in the pre- and post-implementation periods presented according to the diagnoses treated.

### 3.4. Opioid Utilization for Patients Recieving ED-ALTO Procedures

**Figure 1:**
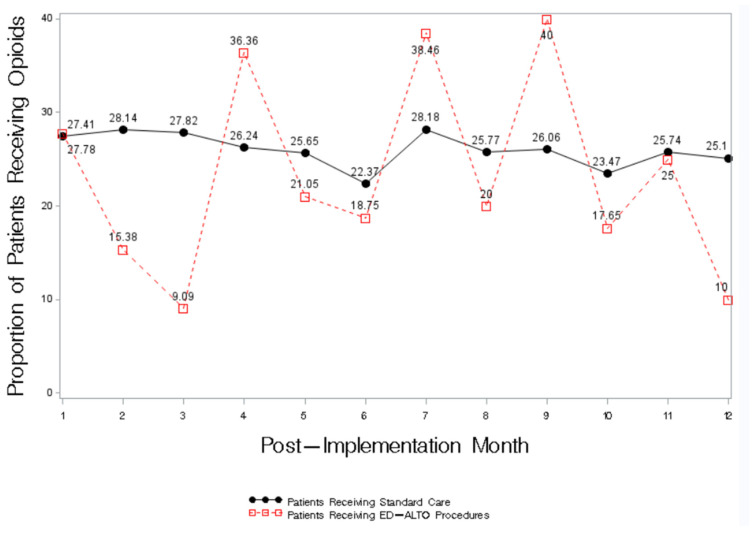
Proportion of Patients Receiving an Opioid prescription in the ED According to ED-ALTO Procedure Status.

Compared to the patients with the same diagnoses who received standard care, in 8 of the 12 months following ED-ALTO’s implementation, a lower proportion of patients receiving ED-ALTO procedures received an opioid in the ED (Figure 1). In months 2, 3, 10, and 12, a marked reduction in the proportion of patients receiving an opioid was observed for the patients receiving an ED-ALTO procedure.

**Figure 2:**
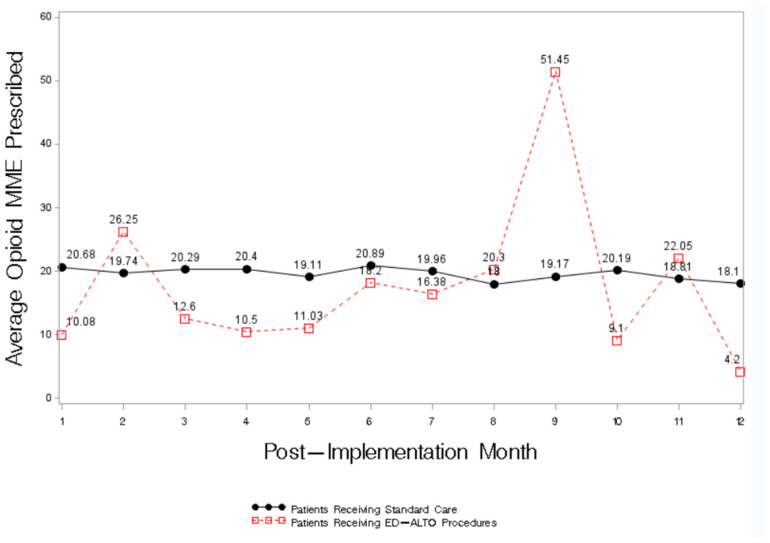
Average Opioid MME prescription by ED-ALTO Procedure Status.

Similarly, compared to the patients with the same diagnoses who received standard care, the patients who received an ED-ALTO procedure had lower average opioid MME in 8 of the 12 months after the study’s implementation (Figure 2).

## 4. Discussion

Our results reflect the first-year results from the implementation of a multi-faceted ED-ALTO program carried out in the Southeastern United States. Our ED-ALTO program includes expanded interventions including both medications and procedure-based approaches to treating pain in the ED. Following implementation, we demonstrated an overall ED-level decrease in opioid utilization with a concomitant increase in the use of ED-ALTO medications and procedures. Among the patients receiving ED-ALTO procedures, in 8 of the 12 months following the program’s implementation, opioid utilization and dosage was lower compared to patients with similar conditions receiving standard care.

The ED-ALTO programs initiated within other institutions in the United States have focused on medication-based changes to the treatment of pain [7,8,9,10,11]. Given that most of the medications were not novel with respect to ED care prior to this intervention, we did not anticipate a large increase in ED-ALTO medication use, yet our results do show a small overall increase in medication utilization. Instead, our emphasis was primarily on providers’ training and uptake of novel procedures to treat pain. We extended the use of ED-ALTO procedures that had never been previously used in the ED setting and expanded the range of conditions that could be treated by ED-ALTO procedures in the ED. Although there was variability, we saw a trend for lower overall opioid use and dosage among those patients receiving ED-ALTO procedures compared to similar patients treated with standard ED pain treatment regimens.

Our program encouraged providers to implement ED-ALTO alternatives as a first-line treatment for varied pain complaints. Providers were encouraged to start with ED-ALTO alternatives during the initial patient evaluation, which might have occurred while waiting for diagnostic imaging and reports. The majority of ED-ALTO interventions were applied for appropriate, expected diagnoses. However, the push to initiate the treatment of symptomatic pain, as opposed to a final diagnosis, did result in some cases in which final diagnoses might be considered inappropriate for alternative treatments. However, all interventions were applied in a timely manner without the delay of other care, including opioids when needed. In many cases, patients’ pain was successfully treated with ED-ALTO interventions that have a very low risk of complications.

We observed that among the patients receiving an opioid, the average MME increased in the post-implementation period. One potential explanation for this finding is that as an increased number of lower-acuity patients are treated with ED-ALTO procedures and medications, the remaining pool of patients presenting with pain will be comprised of patients with more severe conditions and injuries that require higher opioid dosage. Currently, the ED-ALTO protocols are focused on decreasing the widespread and often indiscriminate use of opiates in lower acuity patients (ESI 3-5). Higher-acuity patients were not eligible for this study. However, the success of ED-ALTO therapies for lower-acuity patients suggests that some of these approaches may be applicable to higher-acuity patient populations as well. In particular, nerve blocks, in the presence of dislocations and fractures, can provide targeted pain relief without the negative side effects associated with opiates.

Our ED-ALTO program includes multi-faceted training with which to create a culture of change within the ED and create sustainable, multi-modal, opioid-free treatment options to help treat various pain complaints commonly cared for in the ED. We adopted a model using “Opioid-free days”, in which bedside support was available from physicians with expertise in pain management and acupuncture. During those “opioid-free days”, we administered bedside training to available physicians. We believe a hands-on training approach is necessary for initiating a change in practice patterns and allowing ED physicians to feel comfortable performing new procedures.

### Limitations

There are several limitations associated with this study. Due to the design of this study, it is possible that the statistically significant reduction in opioid utilization we observed after ED-ALTO’s implementation may be due to overall historical trends and the national focus on decreasing opioid use. Our current analysis cannot isolate the effect of our ED-ALTO intervention on opioid practices. Although, we did find an increase in the frequency of ED-ALTO procedures and medication use, which cannot be excluded as a potential factor related to the observed reduction in opioid use. Furthermore, Year 1 of this study occurred during the ongoing COVID-19 pandemic, which created unique challenges for ED research. The COVID-19 pandemic and patient surges often limited ED-ALTO enrollment and eligible patient populations, thus limiting our sample size in Year 1. Furthermore, the overall climate of lockdowns likely prevented certain injuries and exposures, which changed the typical care-seeking patterns and the patients visiting the ED. Additionally, it is possible that the Epic report data used for this analysis may include opioid utilization that was not dispensed in the ED. In Month 9 following ED-ALTO’s implementation, we found that there was one patient with very high opioid utilization. It was later identified that opiate orders were completed in the ED but originated from inpatient orders while a patient was awaiting transport to their final inpatient unit. Therefore, further analysis may reveal an even greater decrease in opioid utilization and MME levels in ED-ALTO-treated patients.

Finally, the unpredictable nature of the complaints presenting to the ED, the pace of care within the ED setting, and the broad ED-ALTO medication order set makes it difficult to attribute all the results regarding the patients receiving ED-ALTO medications to our study efforts. The development of the medication order set was designed to make the prescription of alternative medications more accessible. While it is our intent to increase the use of alternative medications and procedures to treat pain, much of the use of ED-ALTO medications occurred outside of consenting and enrolled patients.

## 5. Conclusions

The opioid crisis continues to be a significant problem in the US, and overdose deaths are on the rise. While pain is a major reason why patients seek care in the ED, it is unnecessary for the successful treatment of pain to be solely focused on the use of opioids, which have many negative side effects. The continued expansion of ED-ALTO programs across the US may serve as a mechanism with which to reduce opioid utilization and successfully help patients control pain. Although our program is in its early stages of implementation, we believe it is showing early success with respect to reducing opioid utilization. We have initiated patient follow-up surveys after ED discharge to assess pain scores for patients receiving ED-ALTO procedures and medications, and we hope to use these data in the future to highlight the effectiveness of ED-ALTO treatments for acute pain in the ED.

## Figures and Tables

**Table 1:** Characteristics of Patients who had Emergency Department Encounters in the Pre- and Post-implementation Period.

	Total Sample	12 Months Pre-Implementation	12 Months Post-Implementation	*p*-Value
	N = 157,457	N = 78,962	N = 78,495	
**Mean Age (SD)**	51.1 (19.9)	51.4 (19.9)	50.8 (19.9)	**<0.01**
**Race ^a^, N (%)**				**<0.01**
White/Caucasian	97,296 (61.8)	49,302 (62.4)	47,994 (61.1)	
Black/African American	42,697 (27.1)	21,335 (27.0)	21,362 (27.2)	
Hispanic	12,926 (8.2)	6153 (7.8)	6773 (8.6)	
Asian	801 (0.5)	413 (0.5)	388 (0.5)	
American Indian or Native Hawaiian	473 (0.3)	239 (0.3)	234 (0.3)	
Other	3264 (2.1)	1520 (1.9)	1744 (2.2)	
Gender, N (%)				0.69
Female	79,810 (50.7)	40,069 (50.7)	39,741 (50.6)	
Male	77,575 (49.3)	38,860 (49.2)	38,715 (49.3)	
Unknown	72 (0.05)	33 (0.04)	39 (0.05)	
**Payor Status, N (%)**				**<0.01**
Public	79,468 (50.5)	39,152 (49.6)	40,316 (51.4)	
Private	41,682 (26.5)	21,136 (26.8)	20,546 (26.2)	
Self-Paid	33,944 (21.6)	17,517 (22.2)	16,427 (20.9)	
Worker’s Comp.	1270 (0.8)	656 (0.8)	614 (0.8)	
Other	1093 (0.7)	501 (0.6)	592 (0.7)	
**Disposition, N (%)**				**<0.01**
Admitted	33,433 (21.2)	17,371 (22.0)	16,062 (20.5)	
Discharged	100,067 (63.5)	50,145 (63.5)	49,922 (63.6)	

^a^ Other race includes participants that listed race as Unknown, Biracial, Refused to answer, or Other, or where such data were missing.

**Table 2:** ED-ALTO medication and opioid utilization pre-and post-implementation.

	Total Sample	12 Months Pre-Implementation	12 Months Post-Implementation	*p*-Value
	N = 157,457	N = 78,962	N = 78,495	
**ED-ALTO Medication Utilization**	53,639 (34.1)	26,568 (33.6)	27,071 (34.5)	**<0.01**
**Received any opioid, N (%)**	26,947 (17.4)	13,968 (17.7)	12,979 (16.5)	**<0.01**
Morphine	13,305 (8.4)	6656 (8.4)	6649 (8.5)	0.77
**Dilaudid**	**3436 (2.2)**	**2140 (2.7)**	**1296 (1.6)**	**<0.01**
Fentanyl	6836 (4.4)	3481 (4.4)	3355 (4.3)	0.19
**Hydrocodone**	**4871 (3.1)**	**2596 (3.3)**	**2275 (2.9)**	**<0.01**
Oxycodone	3660 (2.3)	1802 (2.3)	1858 (2.4)	0.26
Tramadol	550 (0.3)	284 (0.4)	266 (0.3)	0.48
**MME of Opioids ^a^, mean (SD)**	20.8 (21.9)	20.3 (20.3)	21.3 (23.5)	**<0.01**

^a^ Among patients who were prescribed opioids.

**Table 3:** ED-ALTO Alternative Procedure Utilization in the Pre- and Post-implementation Periods by Diagnoses Treated.

	Pre- Period N	Pre-Implementation Diagnoses Treated	Post- Period N	Post-Implementation Diagnoses Treated *
**Total Procedures**	**98**		**167**	
**Nerve Block, N**	**86**		**82**	
Endocrine, Nutritional, Metabolic	1	Type 2 Diabetes	0	
Mental and Behavioral Disorders	1	Alcohol Related Disorders	0	
Diseases of the Nervous System	0		1	**Headache**
Circulatory System	1	Cerebrovascular aneurysm	0	
Digestive System	31	Diseases of the mouth, teeth, and jaw	36	Diseases of the mouth, teeth, and jaw; Other chronic pain
Skin and Subcutaneous Tissue	3	Cellulitis and acute lymphangitis; Nail Disorders	0	
Musculoskeletal and Connective Tissue	6	Chronic lower back pain; Soft tissue disorders	2	Chronic low back pain
Genitourinary System	1	Priapism	0	
Symptoms, Signs and Abnormal Clinical and Lab Findings	2	**Headache**; Syncope and collapse	4	**Headache**; Jaw Pain
Injury, Poisoning, Certain Other Consequences of External Causes	35	Injury of the lower arm; Injury of the hand, wrist, or finger(s); Injury of the knee; Injury of the foot, ankle, or toe(s); Injury (unspecified)	36	Injury of the skull and facial bones; **Fracture of rib(s)**; **Injury of the shoulder or upper arm**; **Fracture of femur**; Injury of the hand, wrist, or fingers; Injury of the foot, ankle, or toe(s); Poisoning
External Causes of Morbidity	4	Pedestrian conveyance accident; Sprain of thoracic spinal ligament(s); Fall; Assault; Perpetrator of assault	2	Fall; Contact with rodent
Factors Influencing Health Status and Contact with Health Services	1	Orthopedic aftercare	1	Other specified health status
**Trigger Point Injection, N**	**12**		**46**	
Diseases of the Nervous System	1	Carpal tunnel syndrome	1	**Headache**
Circulatory System	0		1	Deep vein thrombosis
Diseases of the Respiratory System	0		1	Asthma
Skin and Subcutaneous Tissue	0		1	Disorders of skin
Musculoskeletal and Connective Tissue	11	**Chronic low back pain**; **Disorders of muscle**; Disorders of bone	32	Disorders of joints; **Chronic Low back pain**; **Disorders of muscle**; **Soft tissue disorders**
Symptoms, Signs and Abnormal Clinical and Lab Findings	0		6	**Headache**; Abdominal pain
Injury, Poisoning, Certain Other Consequences of External Causes	0		4	Injury of thorax; Injury of shoulder or upper arm
**Acupuncture, N**	**0**		**17**	
Diseases of the Nervous System	0		1	Polyneuropathy
Digestive System	0		2	Abdominal Pain
Musculoskeletal and Connective Tissue	0		7	Hip pain; **Chronic low back pain**; **Soft tissue disorders**
Symptoms, Signs and Abnormal Clinical and Lab Findings	0		3	Abdominal Pain; **Headache**
Injury, Poisoning, Certain Other Consequences of External Causes	0		2	Injury of neck; Injury of shoulder or upper arm
External Causes of Morbidity	0		2	Traffic accident; Fall
**Acupuncture and Electrostimulation, N**	**0**		**21**	
Certain Infectious and Parasitic Diseases	0		1	Trichomoniasis
Musculoskeletal and Connective Tissue	0		18	Disorders of joints; **Chronic low back pain**
Symptoms, Signs and Abnormal Clinical and Lab Findings	0		1	Abdominal Pain
External Causes of Morbidity	0		1	Fall
**Acupuncture and Nerve Block, N**	**0**		**1**	
Diseases of the Nervous System	0		1	Disorders of autonomic nervous system
**Trigger Point Injection and** **Electrostimulation, N**	**0**		**2**	
Musculoskeletal and Connective Tissue	0		2	**Chronic low back pain**; Disorders of muscle

* Bolded diagnoses indicate conditions specifically targeted with ED-ALTO education and bedside training.

## Data Availability

Not applicable.

## Abbreviations

ALTO	Alternatives to Opiates
ED	Emergency Department
EHR	Electronic Health Record
ESI	Estimated Severity Index
MME	Morphine Milligram Equivalents
PENS	Percutaneous Electrical Nerve Stimulation
TENS	Transcutaneous Electrical Nerve Stimulation

## Appendix A

**Table A1:** List of ED-ALTO Medications and Procedures.

ED-ALTO Medications	Acetaminophen
	Bupivacaine
	Capsaicin
	Cyclobenzaprine
	Ddavp 40 Mcg Intranasal
	Dexamethasone
	Dextrose
	Diazepam
	Dicyclomine
	Diphenhydramine
	Droperidol
	Gabapentin
	Haloperidol
	Ibuprofen
	Ketorolac
	Lactated Ringers
	Lidocaine 1%
	Lidocaine 1.5 Mg/Kg Iv (Max 200 Mg)
	Lidoderm Patch
	Magnesium
	Marcaine
	Metoclopramide
	Nitrous Oxide
	Normal Saline
	Prochlorperazine
	Promethazine
	Ropivacaine
	Sumatriptan
	Valproic Acid
ED-ALTO Procedures	Acupuncture
	Acupuncture paired with PENS
	Axillary Nerve Block (paired with Suprascapular)
	Erector Spinae Nerve Block
	Fascia Iliaca Nerve Block
	Suprascapular Nerve Block
	Trigger Point Injection

**Table A2:** Disease Label and Associated ICD-10 Codes.

Label	Code
Type 2 diabetes	E11.49
Alcohol Related disorders	F10.920
Headache	Pre: R51.9 Post: G44.201, G44.89, R51.9 (9)
Cerebrovascular aneurysm	I67.1
Diseases of the mouth, teeth, and jaw	Pre: K02.9 (*3*), K03.81, K04.7 (7), K08.89 (18), K13.79 Post: K01.1, K02.9 (4), K04.7 (11), K05.219, K08.409, K08.89 (17), K08.9
Other chronic pain	G89.29
Cellulitis and acute lymphangitis	L03.011, L03.032
Nail Disorders	L60.0
Chronic low back pain	Pre: M52.6, M54.2 (2), M54.81 (2) Post: M52.16, M53.9, M54.12 (3), M54.16, M54.2 (6), M54.40, M54.41, M54.42 (3), M54.50 (12), M54.6 (2), M54.81 (2), M54.9 (4)
Soft Tissue Disorders	Pre: M72.2, M79.2, M79.644, M79.672 Post: M75.22, M79.18, M79.2, M79.652
Priapism	N48.30
Syncope and collapse	R55
Jaw Pain	R68.84
Injury of the lower arm	S52.542A, S56.129A
Injury of the hand, wrist, or fingers	Pre: S61.209A, S60.351A, S60.352A, S61.012A, S61.111A, S61.209A, S61.210A (2), S61.212A, S61.213A, S61.219A, S61.253A, S61.309A, S61.322S, S62.522B, S62.524B, S62.609B, S62.639A, S62.646B, S63.259A, S64.40XA, S68.113A, S68.114A, S68.119A (2), S68.120A, S68.611A, S69.92XA Post: S60.352A, S60.559A, S61.001A, S61.011A, S61.022A, S61.239A, S61.309A (2), S61.319A, S62.522B, S62.630B, S67.10XA, S68.113A, S68.119A, S68.626A, S68.629S, S69.90XA
Injury of the knee	S81.812A
Injury of the foot, ankle, or toe(s)	Pre: S91.112A, S92.302A Post: S82.852A, S91.109A
Injury (unspecified)	T14.90XA (2)
Injury of the skull and facial bones	S02.5XXA, S05.42XA
Injury of the shoulder or upper arm	S42.214A, S43.005A, S43.015A, S46.811A, S46.912A
Injury of thorax	S29.012A (2), S29.019A
Fracture of rib(s)	S22.42XA
Fracture of femur	S72.001A, S72.002A (4), S72.141A, S72.22XA, S72.491A, S72.8X1A
Poisoning	T39.311A
Pedestrian conveyance accident	V00-Y99W27
Fall	Pre: W19.XXXA (2), W19.XXXS, W18.30XA Post: W19.XXXD
Assault	Y04.8XXA
Perpetrator of assault	Y07.50
Contact with rodent	W53.21XA
Sprain of thoracic spinal ligament(s)	.3XXA
Orthopedic aftercare	Z47.89
Other specified health statue	Z78.9
Polyneuropathy	G62.9
Abdominal Pain	K85.20; K92.1; R10.9 (4)
Hip Pain	M25.551
Injury of neck	S16.1XXA
Traffic accident	V87.7XXA
Carpel tunnel syndrome	G56.02
Deep vein thrombosis	I82.622
Asthma	J45.901
Disorders of skin	L98.499
Disorders of muscle	Pre: M62.830 (4), M62.838 (3) Post: M62.830 (5), M62.838 (4)
Disorders of bone	M89.8X1
Disorders of joints	M25.511 (3), M25.512 (4), M25.551, M25.561
Trichomoniasis	A59.9
Disorders of autonomic nervous system	G90.512
